# Spontaneous vesicle recycling in the synaptic bouton

**DOI:** 10.3389/fncel.2014.00409

**Published:** 2014-12-08

**Authors:** Sven Truckenbrodt, Silvio O. Rizzoli

**Affiliations:** ^1^Department of Neuro- and Sensory Physiology, University of Göttingen Medical Center, European Neuroscience Institute, Cluster of Excellence Nanoscale Microscopy and Molecular Physiology of the BrainGöttingen, Germany; ^2^International Max Planck Research School for Molecular BiologyGöttingen, Germany

**Keywords:** spontaneous release, synaptic vesicle recycling, synaptic vesicle pools, synaptic vesicle biogenesis, synapse development

## Abstract

The trigger for synaptic vesicle exocytosis is Ca^2+^, which enters the synaptic bouton following action potential stimulation. However, spontaneous release of neurotransmitter also occurs in the absence of stimulation in virtually all synaptic boutons. It has long been thought that this represents exocytosis driven by fluctuations in local Ca^2+^ levels. The vesicles responding to these fluctuations are thought to be the same ones that release upon stimulation, albeit potentially triggered by different Ca^2+^ sensors. This view has been challenged by several recent works, which have suggested that spontaneous release is driven by a separate pool of synaptic vesicles. Numerous articles appeared during the last few years in support of each of these hypotheses, and it has been challenging to bring them into accord. We speculate here on the origins of this controversy, and propose a solution that is related to developmental effects. Constitutive membrane traffic, needed for the biogenesis of vesicles and synapses, is responsible for high levels of spontaneous membrane fusion in young neurons, probably independent of Ca^2+^. The vesicles releasing spontaneously in such neurons are not related to other synaptic vesicle pools and may represent constitutively releasing vesicles (CRVs) rather than *bona fide* synaptic vesicles. In mature neurons, constitutive traffic is much dampened, and the few remaining spontaneous release events probably represent *bona fide* spontaneously releasing synaptic vesicles (SRSVs) responding to Ca^2+^ fluctuations, along with a handful of CRVs that participate in synaptic vesicle turnover.

## Introduction: the phenomenon of spontaneous release at the synaptic bouton

Neuronal communication relies on precisely timed synaptic vesicle exocytosis, which is typically triggered by a brief intracellular Ca^2+^ spike that follows action potentials (Südhof, 2004; Rizzoli, 2014). However, in parallel to stimulated release, all synapses also display spontaneous release of neurotransmitter. This phenomenon has been observed very early on in the history of research in synaptic communication, starting with several landmark papers quantifying synaptic release under physiological conditions, and typically occurs at very low rates of less than 0.1 Hz (Fatt and Katz, 1950, 1952; Del Castillo and Katz, 1954). This type of release did not receive significant further attention until recently (see reviews by Ramirez and Kavalali, 2011; Andreae and Burrone, 2014; Kaeser and Regehr, 2014; Rizzoli, 2014). The main difference between these two modes of release is that stimulated release is directly coupled to action potentials, while spontaneous release can occur seemingly without any trigger, at any time. Spontaneous release can be increased experimentally, by Ca^2+^-dependent mechanisms, such as caffeine triggered Ca^2+^ release from internal stores (Zefirov et al., 2006), as well as by Ca^2+^-independent mechanisms, including application of lanthanum ions (Heuser and Miledi, 1971), of black widow spider venom, whose main active component is alpha-latrotoxin (Ceccarelli et al., 1972, 1973; Betz and Henkel, 1994), or hyperosmotic sucrose (Rosenmund and Stevens, 1996).

We give here an overview of the phenomenon of spontaneous release, briefly reviewing some of the available evidence on its Ca^2+^ dependency. We then focus on the intense recent debate on whether active and spontaneous release originate from the same pool of vesicles. We suggest a simple solution to this problem: two completely different pools of vesicles share the synaptic bouton—synaptic vesicles, which can respond to stimulation but also act as spontaneously releasing synaptic vesicles (SRSVs) and constitutively releasing vesicles (CRVs), which are involved in constitutive membrane traffic and which are not responsive to stimulation. The prevailing view that we are following in this review is that exocytosis of synaptic vesicles is mainly Ca^2+^-dependent, either in response to action potentials or following spontaneous Ca^2+^ fluctuations. The CRVs only exocytose spontaneously, and may be completely independent of Ca^2+^. Finally, we show that this hypothesis is in agreement with the potential roles for spontaneously releasing vesicles throughout neuronal development.

## The calcium sensors for spontaneous release

As indicated above, there are many ways in which spontaneous release can be elicited from synaptic terminals. The machinery for synaptic vesicle release is clearly prone to release even in the absence of action potentials, and even in the absence of Ca^2+^, both external and internal (Rizzoli and Betz, 2002). Whether physiological spontaneous release is Ca^2+^-dependent has been a matter of debate, and has triggered the search for a specific Ca^2+^ sensor. Synaptotagmin 1 has been recognized as the major Ca^2+^ sensor for mediating synaptic release, but mainly in the context of stimulation (Geppert et al., 1994; Südhof, 2004). However, synaptotagmin 1 has also been proposed to facilitate spontaneous release: knock-ins with enhanced apparent Ca^2+^ affinity increased spontaneous release, while knock-ins with reduced apparent Ca^2+^ affinity decreased spontaneous release (Xu et al., 2009; but also see Littleton et al., 1993). This is in agreement with the hypothesis that physiological spontaneous release is caused by spontaneous Ca^2+^ fluctuations. Paradoxically, however, the knock-out of synaptotagmin 1 does lead to a severe increase in spontaneous release (Xu et al., 2009), suggesting that alternative Ca^2+^ sensors must also play a role in this process. The cytoplasmic high-affinity Ca^2+^ binding protein Doc2b has instead been proposed as a specialized sensor for spontaneous release (Groffen et al., 2006, 2010). The knock-out of Doc2b leads to a severe decrease in spontaneous release, but leaves stimulated release unaltered (Groffen et al., 2010). Mechanistically, the higher affinity for Ca^2+^ of Doc2b could make it responsive to small fluctuations in local Ca^2+^ levels that would be sub-threshold for the facilitation of release via synaptotagmin 1, an effect that is mimicked by synaptotagmin 1 mutants with high affinity for Ca^2+^ (Xu et al., 2009). Such local Ca^2+^ fluctuations might, however, also be high enough to trigger release via activation of synapotagmin 1, and Doc2b might play a different role in spontaneous release, since a Ca^2+^ binding deficient Doc2b mutant restored spontaneous release in knock-downs (Pang et al., 2011).

## Different views on the spontaneous pool of synaptic vesicles

The association of the soluble protein Doc2b with the vesicles containing synaptotagmin 1 would render them prone to both spontaneous and stimulated release (see also Walter et al., 2011). Alternatively, vesicles containing only one of these molecules would release only spontaneously, or only during stimulation. Whether the sensor molecules are present on all vesicles is currently unknown. Their large copy numbers, however (about 15 synaptotagmin 1, and about 10 Doc2 molecules per vesicle, on average; Takamori et al., 2006; Wilhelm et al., 2014), make it probable that most of the *bona fide* synaptic vesicles are associated with at least a few copies of both.

**Figure 1:**
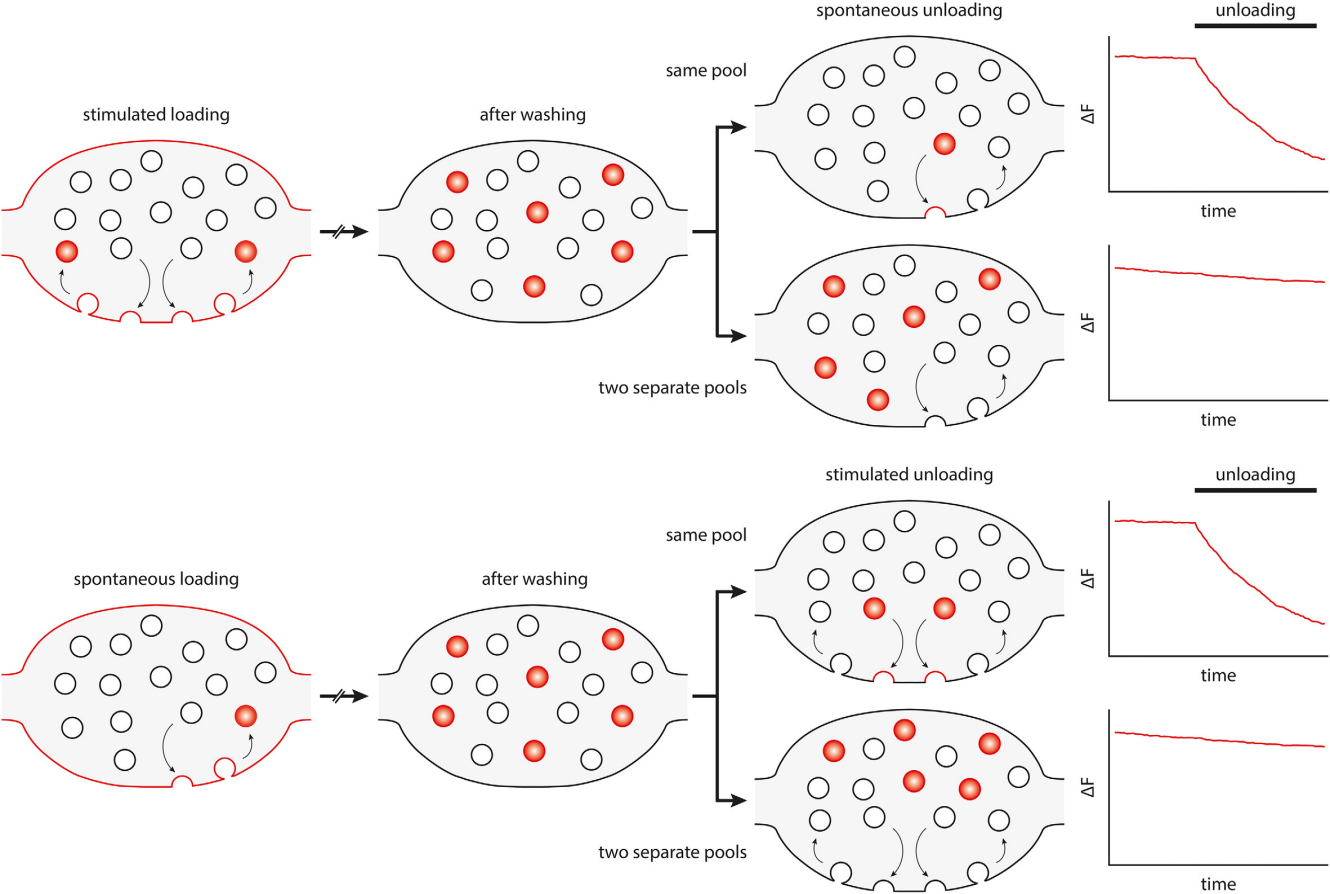
**Principle and interpretation of FM dye loading/unloading experiments that have been used to differentiate spontaneous and active vesicle recycling**. FM dyes partition into the lipid bilayer without crossing it. The dye is taken up into vesicles recycling under stimulation (upper panel) or under spontaneous conditions (spontaneous loading, lower panel). The dye is then washed out from the bath, to follow only loaded vesicles. Subsequently, their unloading behavior is studied by measuring the fluorescence loss, either under stimulation or in spontaneous release conditions. The bouton diagrams and the intensity graphs show, in an idealized fashion, what would happen when the same pool of vesicles participates in both spontaneous and active recycling (upper diagrams), or when the two pools are separate (lower diagrams).

The debate of whether spontaneous release occurs from the same pool of synaptic vesicles as stimulated release has been complicated by many conflicting data published in recent years (for example, Sara et al., 2005; Groemer and Klingauf, 2007; Mathew et al., 2008; Fredj and Burrone, 2009; Wilhelm et al., 2010). Spontaneous recycling has been recently investigated in neuronal cultures by silencing stimulated activity with tetrodotoxin (TTX), which abolishes action potentials. Under these conditions, synaptic vesicles participating in spontaneous release and recycling can be selectively loaded with membrane dyes, such as the FM dyes. After washing out the dye, the exocytosis of the FM-loaded vesicles can be monitored by measuring the loss of fluorescence, as the dye is released from the vesicles into the bathing fluid (see Figure 1).

This type of experiment has initially suggested that vesicles loaded with dye under spontaneous conditions could not be induced to unload by stimulation, and, conversely, that synaptic vesicles loaded with dye during stimulation were not released spontaneously, in TTX (Sara et al., 2005). These observations were later verified by further FM dye experiments (Mathew et al., 2008; Chung et al., 2010). Surprisingly, other groups performed the same experiments, under comparable conditions, and found the opposite: namely that the same vesicles could recycle both under spontaneous and stimulated conditions (Groemer and Klingauf, 2007; Wilhelm et al., 2010).

One possible explanation for this controversy was that the FM dye experiments were difficult to analyse, and that the conclusions depended strongly on the methods used for quantification and normalization (Groemer and Klingauf, 2007). Several other types of labeling were therefore employed, ranging from the enzymatic coupling of biotin to synaptic vesicle proteins, to be later detected by fluorophore-coupled streptavidin (Fredj and Burrone, 2009), to synaptotagmin 1 labeling by antibodies recognizing its intravesicular tail (Hua et al., 2010; Wilhelm et al., 2010). These experiments served to deepen the controversy, by providing evidence both for a separate vesicle pool driving spontaneous release (Fredj and Burrone, 2009), and for the opposite, two independent pools (Hua et al., 2010; Wilhelm et al., 2010).

## The role of spontaneous release during development suggests a simple solution for the problem of the spontaneous pool

How can these conflicting results be reconciled? The key to understanding spontaneous release may lie in its likely role in synaptic biology. Spontaneous release has often been dismissed as a purely stochastic phenomenon, an accidental fusion of synaptic vesicles which should normally only fuse in response to a stimulus. This view is probably incorrect. The machinery of synaptic release has evolved to restrict accidental release of neurotransmitter, with various levels of fail-safe mechanisms to prevent accidental fusion (Jahn and Fasshauer, 2012; Südhof, 2013). At the same time, there is mounting evidence that spontaneous release is far from useless to the neuron, and that it actually has a crucial role in synapse biogenesis, maturation, and maintenance (McKinney et al., 1999; Saitoe et al., 2001; Sutton et al., 2006; Choi et al., 2014; Kaeser and Regehr, 2014).

First of all, we need to consider how synapses and synaptic vesicles are formed. The establishment of synapses entails the transport of various building blocks (for the active zone, the cytoskeleton, synaptic vesicles, and cell–cell adhesion complexes) from the cell soma to the site of the newly forming synapse. These building blocks are transported via various carriers, such as the so called piccolo–bassoon transport vesicles (PTVs) (Zhai et al., 2001; Shapira et al., 2003), or synaptic vesicle protein transport vesicles (STVs) (Sabo et al., 2006). Synaptic vesicles themselves are probably composed of proteins coming from different transport organelles and are later fully assembled in the synaptic bouton itself (see, for example, Rizzoli, 2014). Although various theories for synapse formation exist (see, for example, Scheiffele, 2003; McAllister, 2007), presynaptic STV clustering and release of neurotransmitter are essential, and may even predate the assembly of most other elements of the synapse (Sabo et al., 2006; see also Kaeser and Regehr, 2014).

Since the highly specific machinery of the active zone and *bona fide* synaptic vesicles are still missing from the to-be synapse, both neurotransmitter release and building block delivery must be ensured by CRVs. Such organelles are known from all cell types, where they typically participate in the early (sorting) endosome pathway. They have been described in nascent synapses as a heterogeneous population of dense-core, tubulovesicular, and pleomorphic vesicles, ranging in size up to ~80 nm (Ahmari et al., 2000; Zhai et al., 2001; Ziv and Garner, 2004). This view is consistent with the observation that spontaneous neurotransmitter release is especially prevalent at developing synapses, where it is largely independent of stimulation (Kraszewski et al., 1995; Coco et al., 1998) and accounts for up to 80% of all release (Andreae et al., 2012). It should be noted in this context that constitutive fusion of vesicles outside active zones, and even outside synapses, where it cannot be stimulated by Ca^2+^, is a common observation, especially in developing neurons (Hume et al., 1983; Young and Poo, 1983; Matteoli et al., 1992; Zakharenko et al., 1999; Sabo and McAllister, 2003). Furthermore, spontaneous release and synapse formation are not impaired in knock-outs of synaptotagmin 1, the main Ca^2+^ sensor of synaptic vesicles responsive to stimulation (Geppert et al., 1994). Additionally, synapse formation is not impaired in Doc2a/b double knock-outs (Groffen et al., 2010). Thus, it is tempting to hypothesize that in developing synapses spontaneously fusing vesicles are not *bona fide* SRSVs, but CRVs. As the synapses become established, stimulated release becomes predominant, and spontaneous release is dampened, consistent with the reduced need to deliver fresh building blocks to the synapse (Andreae et al., 2012).

**Figure 2:**
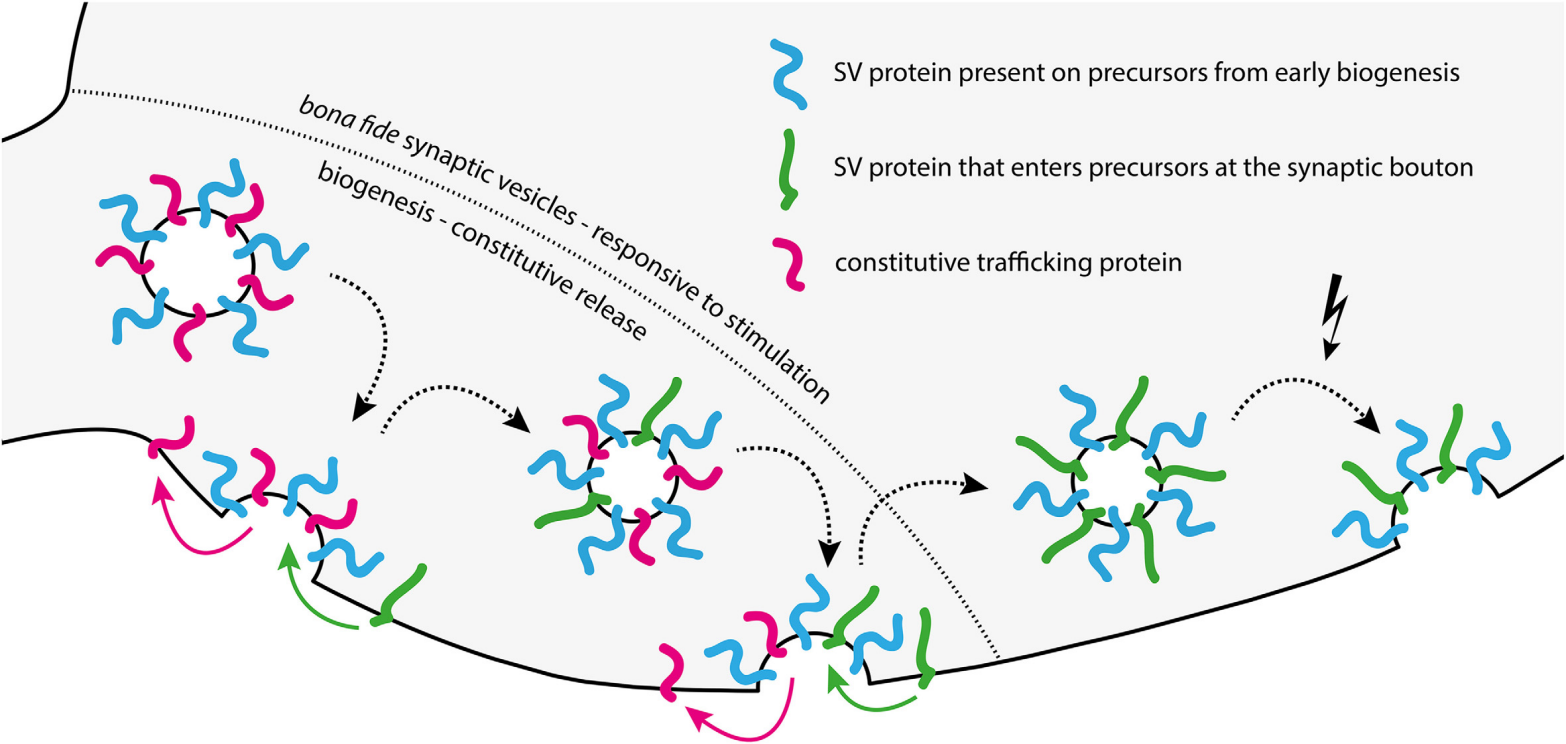
**Model of changes in synaptic vesicle composition and release behavior during biogenesis**. Biogenesis from left to right: CRVs arriving at the synaptic bouton are molecularly “messy” synaptic vesicle precursors, having a different protein composition than *bona fide* synaptic vesicles. These precursors release spontaneously using constitutive trafficking proteins left over from biogenesis in the ER/Golgi system. They may lack the machinery necessary to sense and respond to stimulation. After several rounds of spontaneous release and recycling in the synapse, the emerging vesicle is cleared of most constitutive release proteins left over from biogenesis, and takes up additional synaptic vesicle (SV) proteins. With each recycling step during this phase, the vesicle becomes a bit less competent for spontaneous release, but a bit more competent for stimulated release. The end result is a fully formed, *bona fide* synaptic vesicle with little left over contamination (right of the dashed line).

The nature of the CRVs is not yet fully clear. A recent study suggested that they contain some synaptic vesicle markers, such as synaptophysin and neurotransmitter transporters, but have significantly lower amounts of some other proteins, most significantly the calcium sensor synaptotagmin 1, when compared to *bona fide* synaptic vesicles. At the same time, CRVs were enriched in proteins of endosomal and constitutive trafficking pathways, such as the SNAREs syntaxin 13 and VAMP4 (Revelo et al., 2014). There are at least three different possible explanations for this observation. First, these vesicles might represent an organelle that is, in terms of biogenesis, completely separate from *bona fide* synaptic vesicles, and is involved in, for example, delivery of membrane to the synaptic bouton (unlikely, since these vesicles do contain synaptic vesicle markers). Second, CRVs might represent “rejects” from the synaptic vesicle biogenesis pathway. It is conceivable that biogenesis will not be successful for each and every synaptic vesicle, especially considering the complexity of the trafficking and final assembly steps involved in synaptic vesicle biogenesis (Bonanomi et al., 2006; Rizzoli, 2014). The CRVs might be faulty synaptic vesicles that have the ability to accumulate neurotransmitter (since otherwise there would not be any postsynaptic response to be observed), but without sufficient amounts of Ca^2+^ sensors for stimulated release, or inhibitors of spontaneous release (synaptotagmin 1, see above). Conversely, the insufficient clearing of constitutive and endosomal trafficking proteins from CRVs could thus enable them to release neurotransmitter independent of any stimulation, in a constitutive manner. Third, CRVs may not be “rejects” of the biogenesis pathway, but rather intermediates in the same pathway. The ability to release spontaneously might represent a transitory stage in the life cycle of a synaptic vesicle, during which it sheds unnecessary proteins, such as those of the constitutive pathway, and enriches synaptic vesicle proteins, via repeated fusion with the plasma membrane and subsequent recycling (Rizzoli, 2014). Thus, synaptic vesicles may fuse spontaneously early during their biogenesis, and stop doing so later, as they lose molecules of the constitutively trafficking pathway, and enrich *bona fide* synaptic vesicle molecules (Figure 2).

This third model also provides an explanation for the conflicting results obtained by several groups in the FM dye experiments relating to spontaneous release. If the CRV is an intermediate in the synaptic vesicle biogenesis pathway, it is probable that it still lacks some of the proteins necessary for stimulated release. Specifically, CRVs seem to contain lower levels of VAMP2 and synaptotagmin 1 (Revelo et al., 2014). This would render them less competent to respond to stimulation than *bona fide* synaptic vesicles, or even completely incompetent (see, for example, Rizzoli and Betz, 2002). Since a comparison of seven key studies on spontaneous release makes it evident that virtually identical experimental paradigms often yielded opposing results (Supplementary Table 1), we suggest the following explanation. It is well known that neuronal cultures of the same age can nevertheless have vastly different maturation rates, strongly dependent on, among other details, plating density (Fletcher et al., 1994; Biffi et al., 2013). This varies between laboratories, so despite the similar ages of the cultures, the different studies may have used cultures with different maturation states, and therefore with different rates of constitutive trafficking.

In line with this, studies suggesting one single pool are often based on tools specifically targeting synaptic vesicle proteins, such as VAMP2 or synaptotatmin 1 (see Supplementary Table 1) (Hua et al., 2010; Wilhelm et al., 2010). CRVs seem to contain less of these markers (Revelo et al., 2014), and therefore cannot be efficiently labeled this way, while FM dyes are non-specific and can label CRVs and SRSVs. The SRSVs would be releasable via stimulation, while the CRVs would not. Most studies discerning a separate spontaneous pool are based on FM dyes and find at least some unloading from this pool under stimulation (Sara et al., 2005; Mathew et al., 2008; Chung et al., 2010). The “spontaneous pool” labeled in FM-experiments was thus probably heterogeneously composed of CRVs and SRSVs. CRV prevalence decreases with neuron maturity (Andreae et al., 2012), preventing the detection of a separate spontaneous pool.

## Molecular markers of the spontaneously recycling vesicles

Several recent studies found that vesicles containing the overexpressed SNAREs VAMP7 (Hua et al., 2011; Bal et al., 2013) or Vti1a (Ramirez et al., 2012) behaved like CRVs, and were not responsive to stimulation. Since these molecules are classical constitutive trafficking markers, it is not surprising that they reach a type of vesicles that is not equivalent to synaptic vesicles. VAMP7 has been implicated in growth cone development and late endosome/lysosome trafficking (Wang and Tang, 2006; Burgo et al., 2012), while Vti1a is known to participate in cis- and trans-Golgi trafficking (Fischer von Mollard and Stevens, 1998; Ganley et al., 2008). Additionally, a VAMP7/Vti1a SNARE complex has been implicated in constitutive exocytosis and potassium channel trafficking to the plasma membrane, both in neuronal and non-neuronal cells (Flowerdew and Burgoyne, 2009).

The experiments in which overexpressed VAMP7 and Vti1a reached spontaneously-, but not actively-releasing vesicles, have been interpreted as proof for their being the molecular mechanism that controls the reserve pool of synaptic vesicles (see Rizzoli and Betz, 2005, for a review on synaptic vesicle pools). This largely inert pool of vesicles, which does not recycle under physiological stimulation, was seen as the source of spontaneous release, via SNAREs such as VAMP7 (Fredj and Burrone, 2009; Hua et al., 2011; Bal et al., 2013) or Vti1a (Ramirez et al., 2012). In view of more recent evidence, this is highly unlikely. Such molecules are present in mature synapses in very low copy numbers, two to three orders of magnitude lower than the exocytotic SNAREs (Takamori et al., 2006; Wilhelm et al., 2014). Such copy numbers would not allow them to be present in all reserve vesicles (Vti1a averages at only ~1 copy per four vesicles in mature synapses; Wilhelm et al., 2014). Another argument against the hypothesis that VAMP7 and Vti1a drive spontaneous release from the reserve pool is that there is very little spontaneous release in mature synaptic boutons (Andreae et al., 2012), although the reserve pool consists of at least ~50% of all vesicles in mature synapses (Rizzoli, 2014). In contrast, the low copy numbers of SNAREs of the constitutive pathway agree with the hypothesis that spontaneous release is, at least in developing synapses, performed by constitutively recycling vesicles. Since such vesicles are not present in large numbers in mature synapses, neither should be their characteristic SNAREs.

## Conclusions: what is the purpose of spontaneous release?

We suggest that CRVs are, during the early development of the neuron, a pool of constitutively recycling membranes, which participate in the formation of synapses and synaptic vesicles. Putting this in perspective, a recent study determined that synapse development is perturbed by the abolishment of spontaneous, but not stimulated release (Choi et al., 2014). But even the established synapse is critically dependent on spontaneous neurotransmitter release (as recently reviewed by Andreae and Burrone, 2014). Abolishing it will first induce a compensatory receptor synthesis in the postsynapse (Sutton et al., 2006), later a loss of postsynaptic glutamate receptors (Saitoe et al., 2001), and will ultimately result in dissolving the synapse, evidenced by the loss of the dendritic spine (McKinney et al., 1999). Whether these spontaneously releasing vesicles are still members of the CRV pathway, or whether they are mature SRSVs responding to spontaneous Ca^2+^ fluctuations, perhaps via Doc2b, still remains to be determined.

### Conflict of interest statement

The authors declare that the research was conducted in the absence of any commercial or financial relationships that could be construed as a potential conflict of interest.
