# Supplementary material for: Spontaneous vesicle recycling in the synaptic bouton
Source: Front Cell Neurosci. 2014 Dec 8;8:409. doi: 10.3389/fncel.2014.00409 (PMC4259163; doi:10.3389/fncel.2014.00409)
Supplement: Supplementary file 1 [file Table1.PDF]

Table 1: Experimental parameters in seven studies on the relationship between spontaneous and evoked release.

				stimulated labelling				spontaneous labelling	
study/result	preparation	age	probe	frequency	high K^+	loading time	resting time	loading time	resting time
Sara et al. (2005)<br><br>two separate pools	hippocampal culture	prepared from p2-p3 Sprague-Dawley rats, used after 15-25 days in culture	FM 2-10	30 Hz (or: network activity of the culture)	47 mM (one experiment with physiological 4 mM gave similar results)	2 s for electrical stimulation (or: 15 min for network activity), 90 s for high K^+	10 min	10-45 min in 1 µM TTX, 50 µM AP5, 10 µM CNQX	10 min
Groemer & Klingauf (2007)<br><br>one single pool	hippocampal culture	prepared from p2-p3 Sprague-Dawley rats, used after 15-21 days in culture	FM 5-95 (for stimulated loading), FM 1-43 (for spontaneous loading)	30 Hz	n/a	4 s (+ 90 s resting time with dye)	~26 min (including 10 min of sequential spontaneous loading)	10 min in 1 µM TTX	~8 min
Mathew et al. (2008)<br><br>two separate pools	acute slices (prefrontal cortex; inhibitory neurons)	18-25 days old rats, slices used after 1 h	FM 1-43	10 Hz	40 mM	3.5 min (90 s stimulation after 60 s) for electrical stimulation, 45 s for high K^+	30 min	15 min in 1 µM TTX	30 min
Fredj & Burrone (2009)<br><br>two separate pools	hippocampal culture	prepared from e18 Sprague-Dawley rats, used after ~14 days in culture	"biosyn" (biotinylated VAMP2, detected with fluorescently labelled streptavidin)	n/a	60 mM (together with 1 µM TTX, 25 µM AP5, 20 µM CNQX)	2x 90 s (separated by 5 min resting period)	20 min (including 15 min of sequential spontaneous loading)	15 min in 1 µM TTX, 25 µM AP5, 20 µM CNQX	n/a (fixation directly after wash)
Chung et al. (2010)<br><br>two separate pools	hippocampal culture	prepared from p2-p3 Sprague-Dawley rats, used after 14 days in culture	FM 2-10, FM 1-43, FM 5-95	n/a	47 mM	90 s	10 min	10 min in 1 µM TTX, 50 µM AP5, 10 µM CNQX	10 min
Hua et al. (2010)<br><br>one single pool	hippocampal culture	prepared from p1-p2 Wistar rats, used after 14-21 days in culture	biosyn (see Fredj & Burrone, 2009), synaptotagmin lumenal domain antibodies	20 Hz	n/a	45 s (+ 5 min resting time with dye)	not stated	15-20 min in 1 µM TTX	not stated
Wilhelm et al. (2010)<br><br>one single pool	hippocampal culture; NMJ of mouse, frog and *Drosophila*	prepared from p1-p2 Wistar rats, used after 10 days in culture	FM 1-43, rabbit and mouse synaptotagmin lumenal domain antibodies	20 Hz	n/a	30 s	10 min	15 min in 1 µM TTX	10 min
